# YAP1 and P53 Expression in Papillary Thyroid Carcinoma

**DOI:** 10.30699/IJP.2023.553716.2897

**Published:** 2023-03-23

**Authors:** Dalia Nabil Abdelhafez, Maram Mustafa Ayoub, Samira A. Mahmoud, Hala M. El Hanbuli

**Affiliations:** Department of Pathology, Faculty of Medicine, Fayoum University, Egypt

**Keywords:** Papillary thyroid carcinoma, p53, YAP1

## Abstract

**Background & Objective:**

One of the most prevalent endocrine system cancers is papillary thyroid carcinoma, with complicated predisposing factors and pathogenesis. YAP1 (Yes-associated protein 1) is a well-known oncogene; its activity is increased in a variety of human malignancies and has recently been paid great attention. The present study examines YAP1 and P53 immunohistochemical expression in papillary thyroid carcinoma and investigates the association of their expression with the available clinicopathological risk factors to assess their possible prognostic role.

**Methods:**

The current study used paraffin blocks of 60 cases of papillary thyroid carcinoma, which were immunohistochemically assessed for YAP1 and p53 expression. The study examined the association of their expression with clinicopathological characteristics.

**Results:**

YAP1 expression was observed in 70% of papillary thyroid carcinoma cases. A statistically significant relation was observed between YAP1 expression and tumor size, tumor stage, tumor focality, lymph node metastases, and extrathyroidal extension (P-values were =0.003, > 0.001, 0.037, 0.025, and 0.006), respectively. p53 expression was observed in 85% of papillary thyroid carcinoma cases. A statistically significant relation was observed between p53 expression and tumor size (*P*=0.001) and tumor stage (*P*>0.001). A statistically significant relation was noticed between YAP1 and P53 expression (*P*=0.009).

**Conclusion:**

YAP1 expression was found to be associated with many high-risk clinicopathological characteristics in patients with papillary thyroid carcinoma and with p53 expression; thus, it seems that YAP1 may have a specific impact on the patient's outcome.

## Introduction

Thyroid cancer is the most prevalent endocrine tumor, accounting for 1% of all newly diagnosed cancers (1). The incidence and frequency of thyroid cancer in Egypt are similar to those in Western countries, as it accounts for 1.5 % of all malignancies representing 30% of all endocrine tumors. In Egypt, the thyroid cancer rate is 0.0027% in females, with a female predominance (2).

Papillary thyroid carcinoma (PTC) is the most common type of thyroid cancer (3). It accounts for roughly 80% of all thyroid cancer patients (4). The prevalence of PTC is increasing. The reasons are unknown but may reflect advancements that help earlier cancer detection (5). PTC can develop at any age and is only rarely found as a congenital tumor (6). It is commonly detected in the third to fifth decades of a patient's life, with an average age of 40. The prevalence of PTC rises with age, and women-to-men ratios ranging from 2:1 to 4:1 (7).

The main transcriptional regulator in the Hippo-signaling pathway is Yes-associated protein-1 (YAP1). The major function of this pathway is to control organ size by limiting cell proliferation and increasing apoptosis (8). YAP1 moves between the nucleus and the cytoplasm. When the Hippo pathway is activated, LATS1/2 kinases phosphorylate and sequestrate YAP1 in the cytoplasm, preventing its translocation to the nucleus, thereby promoting transcription of Hippo pathway genes, which are primarily responsible for the proliferation and migration of cells (9). YAP has been identified as an oncogene; its overexpression has been noticed in a variety of human malignancies, including hepatocellular carcinoma, gastric cancer, colorectal cancer, non-small-cell lung cancer, and small-cell lung cancer (10). However, there have been very few reports concerning YAP1 expression in PTC.

TP53 is a tumor suppressor gene that genes for the protein p53, which is involved in cell cycle arrest in damaged cells requiring DNA repair or initiating apoptosis in cells damaged beyond repair. A TP53 mutation is a critical event in carcinogenesis (11), as it is one of the most regularly mutated genes in tumors (12). Various causes of functional p53 inactivation have been described, the majority of which are caused by chromosome 17p deletions (13).

YAP1 and p53 pathways are vital protectors of genomics in response to DNA damage. Their co-expression has never been explored in thyroid cancer. The present study examined YAP1 and p53 expressions in PTC and assessed the associations of their expressions with clinicopathological risk factors, thus assessing whether YAP1 and p53 could be possible prognostic markers.

## Material and Methods

**Tissue Samples**

The present study was conducted on 60 formalin-fixed paraffin-embedded specimens from patients diagnosed with papillary thyroid carcinoma. Specimens were obtained from the archives of the pathology department, Faculty of Medicine, Cairo University, Egypt. Exclusion criteria were preoperative chemo- or radiotherapy prior to surgery. Available clinical data were collected from archival reports. Ethical approval for this study was granted by the Local Ethics Committee.

**Histopathological and Immunohistochemical Staining**

One μm thick tissue section were cut from formalin-fixed paraffin-embedded tissue blocks (3 sections were obtained from each block). One section was stained with routine Hematoxylin and Eosin stain (H&E) for histopathological evaluation. The other two sections were mounted on positively charged sides. After deparaffinization and rehydration, immunohistochemistry (IHC) was performed using a Ventana Discovery XT Automated Slide Stainer. The slices were incubated with primary antibodies against YAP1 (1:200, clone (G-6), Santa Cruz Biotechnology, Dallas, Texas, USA) and p53 (1:1000, clone DO-7; Santa Cruz Biotechnology, Dallas, Texas, USA). The appropriate positive and negative controls were included.

**Immunohistochemical Assessment**

In the present work, immunostaining was evaluated by three independent pathologists. YAP1 nuclear expression was minimal and thus was ignored, and only cytoplasmic staining was considered (14). YAP1 cytoplasmic expression was graded according to the intensity and percentage of positive cells. Negative staining was rendered to the complete absence of reactivity. Weak cytoplasmic reactivity regardless of the extent and strong cytoplasmic reactivity with less than 50% positive cells were graded as low expression. Otherwise, it was graded as a high expression (15). Because of the relatively small sample size and statistical reasons, low and high YAP1 stainings were classified as YAP1 positive expressions.

The nuclear expression of p53 was assessed by determining the percentage of positive cells stained in each tissue section as follows, Negative if there was no nuclear staining in the examined tumor cells or there was nuclear staining in ≤ 5% of cells, Positive if there was nuclear staining in >5% of cells (16).

**Statistical Analysis**

It was performed using SPSS version 16 (SPSS Inc., Chicago, Ill., USA). Variables were presented using number and percent for qualitative variables, median, Mean, standard deviation, and range for quantitative variables. Comparison between groups was made using the chi-square test for qualitative variables and Student's t-test for quantitative variables. Agreement between procedures was tested. A P-value equal to and less than 0.05 was considered statistically significant**.**

## Results

**Fig. 1:**
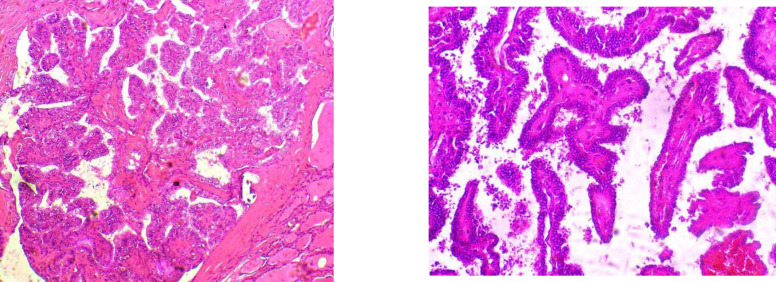
Papillary thyroid carcinoma (H&E); (A) X10, (B) X40

Of the total 60 cases examined, eleven patients (18.3%) were male, and 49 were female (81.7%). Patients aged below 45 years and 45 years and above accounted for 48.3% and 51.7%, respectively. According to tumor size, patients were divided into three groups, Tumors < 2 cm in maximal diameter were found in 17 (28.3%) patients, Tumors size of 2-4 cm in maximal diameter were found in 25 (41.7%) patients and tumors > 4 cm were found in 18 (30%) patients. Among the studied cases; 16 (26.7%) cases presented as stage pT1, 14 (23.3%) cases as pT2, and 30 (50%) cases presented as pT3. Lymph node metastasis was observed in 16.7% of patients. The extrathyroidal extension was noted in 23.3% of patients. Most of the studied cases (70%) were unifocal lesions, while (30%) were multifocal lesions. Different histological types were encountered. There were 41 (68.3%) cases diagnosed as conventional PTC (Figure 1), 8 (13.3%) as follicular variants, 6 (10%) as microcarcinomas, 3 (5%) as oncocytic variants, while each of solid and hobnail variants were represented by 1 case (1.7%).

**Fig. 2:**
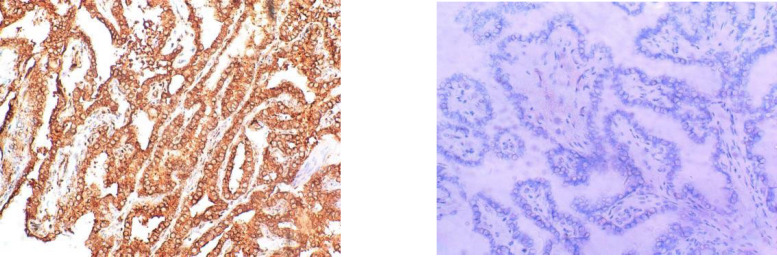
Immunohistochemical expression of YAP1 in PTC; (A) positive cytoplasmic staining X10, (B)negative staining X40

YAP1 expression was positive in 42/60 tumors (70%) and negative in 18/60 tumors (30%) (Figure 2). A statistically significant relation was observed between YAP1 expression and tumor size (*P*=0.003), tumor stage (*P*>0.001), tumor focality (*P*=0.037), lymph node metastasis (*P*=0.025) and extra-thyroidal extension (*P*=0.006). None of the other clinicopathological characteristics were associated with YAP1 expression (Table 1).

**Table 1:** PTC patients' Characteristics According to the immunohistochemical expression of YAP1

Clinicopathological Features	YAP1 expression		*P*
	Positive N (%)	Negative N (%)	
Age < 45 ≥45	20 (69%) 22 (71%)	9 (31%) 9 (29%)	0.99
Sex Male Female	8 (72.7%) 34 (69.4%)	3 (27.3%) 15 (30.6%)	0.99
Tumor size < 2 2-4 > 4	7 (41.2%) 18 (72%) 17 (94.4%)	10 (58.8%) 7 (28%) 1 (5.6%)	0.003
Tumor stage pT1 pT2 pT3	5 (31.2%) 9 (64.3%) 28 (93.3%)	11 (68.8%) 5 (35.7%) 2 (6.7%)	>0.001
Multifocality Present Absent	16 (88.9%) 26 (61.9%)	2 (11.1%) 16 (38.1%)	0.037
Histological variants Conventional Others	31 (75.6%) 11 (57.9%)	10 (24.4%) 8 (42.1%)	0.164
Extrathyroidal extension Present Absent	14 (100%) 28 (60.9%)	0 (0%) 18 (39.1%)	0.006
LN metastasis Present Absent	10 (100%) 32 (64%)	0 (0%) 18 (36%)	0.025

**Fig. 3:**
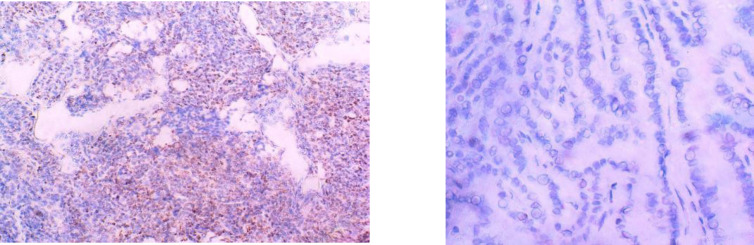
Immunohistochemical expression of P53 in PTC; (A) positive nuclear staining X10, (B) negative staining X40

p53 expression was positive in 51/60 tumors (85%) and negative in 9/60 tumors (15%) (Figure 3). A statistically significant relation was observed between p53 expression and tumor size (*P*=0.001) and tumor stage (*P*>0.001). None of the other clinicopathological characteristics were associated with p53 expression (Table 2).

A statistically significant relation was observed between YAP1 and p53 expression in the studied cases (*P*=0.009).

**Table 2:** PTC patients' Characteristics According to the immunohistochemical expression of p53

Clinicopathological Features	P53 expression		*P*
	Positive N (%)	Negative N (%)	
Age < 45 ≥45	25 (86.2%) 26 (83.9%)	4 (13.8%) 5 (16.1%)	0.8
Sex Male Female	11 (100%) 40 (81.6%)	0 (0%) 9 (18.4%)	0.189
Tumor size < 2 2-4 > 4	10 (58.8%) 23 (92%) 18 (100%)	7 (41.2%) 2 (8%) 0 (0%)	0.001
Tumor stage pT1 pT2 pT3	8 (50%) 13 (92.9%) 30 (100%)	8 (50%) 1 (7.1%) 0 (0%)	>0.001
Multifocality Present Absent	17 (94.4%) 34 (81%)	1 (5.6%) 8 (19%)	0.255
Histological variants Conventional Others	37 (90.2%) 14 (73.7%)	4 (9.8%) 5 (26.3%)	0.126
Extrathyroidal extension Present Absent	14 (100%) 37 (80.4%)	0 (0%) 9 (19.6%)	0.1
LN metastasis Present Absent	10 (100%) 41 (82%)	0 (0%) 9 (18%)	0.146

## Discussion

Papillary thyroid carcinoma is the most common endocrine malignancy, and it has multifactorial predisposing factors. PTC, in general, has an excellent prognosis. The overall 5-year survival rate is 90– 95% (17). Many poor prognostic factors are related to PTC, including older age at diagnosis, male gender, large tumor size, and extrathyroidal growth (7).

Recently, YAP1 (a nuclear effector of an inactivated HIPPO pathway) has been recognized as a potent oncogene closely linked to the prognosis and progression of several types of cancer, including breast and pancreatic cancer (18, 19). In addition, YAP1 has been identified as a tumor marker linked to drug sensitivity in various cancers (20). In colorectal cancer, YAP expression was correlated with tumor progression and prognosis and has been considered by a recent study as an independent predictor of poor prognosis in those patients (21).

Increased expression of YAP1 has also been described in thyroid cancer tissues (22). In particular, Lee* et al.* (23) found that YAP1 was overexpressed in the nucleus and cytoplasm of papillary thyroid carcinoma and anaplastic thyroid cancer, with a higher frequency in patients carrying a V600E BRAF mutation.

In the present study, 70% of PTC expressed YAP1. Nearly similar results were given by Ugolini* et al.* (24), who reported positive YAP1 expression in 70.8% of the studied cases, and Liu* et al.* (25), who reported positive YAP1 expression in 62.1% of the studied cases. Higher results were given by other studies that reported positive YAP1 expression in 95% and 100% of the studied cases (23,26,27). Furthermore, YAP1 expression was significantly related to larger tumor size, advanced tumor stage, tumor focality, lymph node metastasis, and extra-thyroidal extension. The previous study also reported a statistically significant relation between YAP1 expression and larger tumor size, lymph node metastasis, and extra-thyroidal extension (25) and other studies also found a statistically significant relation between YAP1 expression and extra-thyroidal extension (23,24).

Other clinicopathological characteristics studied in this paper were not associated with YAP1 expression, which is going with other previous studies (25,27), with the exclusion of a single study that reported a significant relation between YAP1 expression and gender (24).

p53 is a tumor suppressor inactivated in half of human cancers. So, it has a well-known role in human tumorigenesis (28). Since p53 plays a significant role in DNA damage response, cellular differentiation, proliferation, and death, it represents a very attractive target in anticancer drug development (29).

Alterations in p53 signaling can contribute to thyroid carcinogenesis by displaying checkpoint defects, genomic instability, and inhibition of apoptosis. In addition, p53 tumor suppressor activity is altered in thyroid carcinoma by three different mechanisms that inhibit its transcriptional activity, protein stability, and downstream signaling (30).

Concerning p53 expression in this study, it was expressed in 85% of PTC tumors. These results are close to previous reports (16,31, 32) but are higher than other studies that reported p53 expression in around 40 to 45% of the studied PTC cases (33-36).

Statistical analysis for a possible significant relation between p53 expression and the different clinicopathological variables revealed a statistically significant relation between p53 expression and larger tumor size and more advanced tumor stage. Some of the previous studies showed a similar result (35, 37), while a single study showed a significant relation between p53 expression and smaller tumor size (33) and others showed insignificant relation between p53 expression and tumor size or tumor stage (31,34).

Other studied clinicopathological characteristics were not associated with p53 expression, and this matches previously reported results (31,34) but in contrast to other studies that reported a statistically significant relation between p53 expression and extra-thyroidal extension (33), lymph node metastasis (35), gender (16) absence of multifocal PTC and absence of metastasis (32).

A statistically significant relation was observed between YAP1 and p53 expression in the studied cases going with a recent study that reported YAP1 expression was associated with p53 expression in glioma (38). To the best of our knowledge, this is the first study discussing the association between YAP1 and p53 expression in thyroid cancer. The relationship between the two markers is recently getting attention as some recent studies discussed the reciprocal crosstalk between Hippo and p53 pathways, supposing that YAP1 and mutant TP53 could intensify the proliferative effect of each other, interact in different levels and are closely coordinated (39,40). 

## Conclusion

YAP1 was highly expressed in papillary thyroid carcinoma cases and its expression was significantly related to high-risk clinicopathological features of the patients. Also, p53 was significantly up-regulated in PTC cases, and its expression was associated with tumor progression (tumor size). The expression of YAP1 was significantly associated with that of p53 expression. Both markers can represent potential biomarkers of PTC progression. Further studies on larger samples are recommended to elucidate the role of these markers in the prognosis and progression of PTC lesions.

## Conflict of Interest

The authors declared no conflicts of interest.

## Funding

None.
